# Differences in Susceptibility to SARS-CoV-2 Infection Among Transgenic hACE2-Hamster Founder Lines

**DOI:** 10.3390/v16101625

**Published:** 2024-10-17

**Authors:** Scott A. Gibson, Yanan Liu, Rong Li, Brett L. Hurst, Zhiqiang Fan, Venkatraman Siddharthan, Deanna P. Larson, Ashley Y. Sheesley, Rebekah Stewart, Madelyn Kunzler, Irina A. Polejaeva, Arnaud J Van Wettere, Stefan Moisyadi, John D. Morrey, E. Bart Tarbet, Zhongde Wang

**Affiliations:** 1 Department of Animal, Diary and Veterinary Sciences, Utah State University, Logan, UT 84322, USA; scott.gibson@usu.edu (S.A.G.); yanan.liu@usu.edu (Y.L.); rong.li@aggiemail.usu.edu (R.L.); brett.hurst@usu.edu (B.L.H.); zhiqiang.fan@aggiemail.usu.edu (Z.F.); venkat.siddharthan@usu.edu (V.S.); dpassarolarson@gmail.com (D.P.L.); ashley.sheesley@usu.edu (A.Y.S.); rebekah.stewart@aggiemail.usu.edu (R.S.); madelyn.kunzler@usu.edu (M.K.); irina.polejaeva@usu.edu (I.A.P.); john.morrey@usu.edu (J.D.M.); zonda.wang@usu.edu (Z.W.); 2 Institute for Antiviral Research, Utah State University, Logan, UT 84322, USA; 3 Department of Veterinary, Clinical, and Life Sciences, Utah State University, Logan, UT 84322, USA; arnaud.vanwettere@usu.edu; 4 Utah Veterinary Diagnostic Laboratory, Utah State University, Logan, UT 84322, USA; 5 Institute of Biogenesis Research, John A. Burns School of Medicine, University of Hawaii, Honolulu, HI 96822, USA; moisyadi@hawaii.edu

**Keywords:** SARS-CoV-2, viral pathogenesis, transgenic hamster, animal model

## Abstract

Animal models that are susceptible to SARS-CoV-2 infection and develop clinical signs like human COVID-19 are desired to understand viral pathogenesis and develop effective medical countermeasures. The golden Syrian hamster is important for the study of SARS-CoV-2 since hamsters are naturally susceptible to SARS-CoV-2. However, infected hamsters show only limited clinical disease and resolve infection quickly. In this study, we describe development of human angiotensin-converting enzyme 2 (hACE2) transgenic hamsters as a model for COVID-19. During development of the model for SARS-CoV-2, we observed that different hACE2 transgenic hamster founder lines varied in their susceptibility to SARS-CoV-2 lethal infection. The highly susceptible hACE2 founder lines F0F35 and F0M41 rapidly progress to severe infection and death within 6 days post-infection (p.i.). Clinical signs included lethargy, weight loss, dyspnea, and mortality. Lethality was observed in a viral dose-dependent manner with a lethal dose as low as 1 × 10^0.15^ CCID_50_. In addition, virus shedding from highly susceptible lines was detected in oropharyngeal swabs on days 2–5 p.i., and virus titers were observed at 10^5.5−6.5^ CCID_50_ in lung and brain tissue by day 4 p.i.. Histopathology revealed that infected hACE2-hamsters developed rhinitis, tracheitis, bronchointerstitial pneumonia, and encephalitis. Mortality in highly susceptible hACE2-hamsters can be attributed to neurologic disease with contributions from the accompanying respiratory disease. In contrast, virus challenge of animals from less susceptible founder lines, F0M44 and F0M51, resulted in only 0–20% mortality. To demonstrate utility of this SARS-CoV-2 infection model, we determined the protective effect of the TLR3 agonist polyinosinic-polycytidylic acid (Poly (I:C)). Prophylactic treatment with Poly (I:C) significantly improved survival in highly susceptible hACE2-hamsters. In summary, our studies demonstrate that hACE2 transgenic hamsters differ in their susceptibility to SARS-CoV-2 infection, based on the transgenic hamster founder line, and that prophylactic treatment with Poly (I:C) was protective in this COVID-19 model of highly susceptible hACE2-hamsters.

## 1. Introduction

The emergence and rapid global dissemination of severe acute respiratory syndrome coronavirus 2 (SARS-CoV-2) has resulted in more than 500 million confirmed cases and 6 million deaths worldwide. The most common clinical manifestations associated with coronavirus disease 2019 (COVID-19) in humans include fever, malaise, lethargy, myalgia, sore throat, cough, shortness of breath, and anosmia [1,2,3]. Mortality from SARS-CoV-2 infections in humans has primarily been attributed to severe respiratory disease such as acute respiratory disease syndrome (ARDS) [4]. In addition to respiratory disease, SARS-CoV-2 has demonstrated the ability to infect and damage other organs, including the central nervous system (CNS) [5]. The onset of COVID-19 and its pathogenesis in human patients has been characterized as a complex disease with a wide variety of disease outcomes [6]. This spectrum of outcomes ranges from asymptomatic infection to severe disease that results in hospitalization and death. In addition, lethal cases of SARS-CoV-2 infection can also lead to the development of long-term symptoms (Long COVID-19) [7,8].

An increasing amount of evidence has been reported that suggests a potential link between neurologic symptoms of SARS-CoV-2 infection and the development of severe COVID-19 [9]. Stroke, encephalitis, and hemorrhage are examples of clinical signs reported in lethal cases of COVID-19 that can cause neurologic symptoms [10,11]. The development of psychiatric disorders in humans has also been found to be a neurologic consequence of SARS-CoV-2 infection [12]. Studies in clinical settings have suggested that roughly 1/3 of individuals infected with SARS-CoV-2 have reported some degree of neurological manifestations [13]. Considering the complexities of COVID-19, it is necessary to explore the full range of disease outcomes to better understand the drivers of COVID-19 severity and mortality.

**Figure 1:**
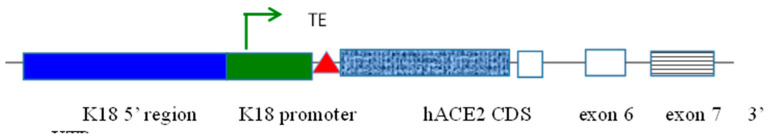
Diagram of epithelium-specific expression cassette for the hACE2 gene, pK18-hACE2.

Infection of wild-type golden Syrian hamsters with SARS-CoV-2 results in modest weight loss and high virus titers in lung tissue with detectable histologic lesions, but the infection does not lead to mortality, so does not model severe SARS-CoV-2 in human patients [14]. Conversely, the human angiotensin-converting enzyme 2 (hACE2) transgenic mouse model is a model of lethal SARS-CoV-2 infection due to neurologic disease with a less significant respiratory component [15,16]. Viral transmission has not been observed in mouse infection models, but transmission does occur in hamster infection models [17]. To generate an alternative lethal infection model, we engineered the hACE2 gene into the golden Syrian hamster genome via piggyBac-mediated transgenesis, whereby the expression of hACE2 is under the control of the human keratin 18 (K18) promoter and other regulatory sequences that are critical for epithelial specificity and produced multiple independent transgenic hACE2-hamster lines (Figure 1).

To ensure epithelium-specific expression, 2.5 kb of the upstream genomic sequence, the promoter, and the first intron of the human K18 gene were placed upstream of the 5′ of the coding sequence (CDS) of the hACE2 gene, and the exon 6, intron 6, exon 7, and the 3′ untranslated region (3′-UTR) of the human K18 gene were placed immediately after the stop codon of the hACE2 gene. For sufficient gene expression of the hACE2 transgene, a translational enhancer (TE) sequence from alfalfa mosaic virus was also cloned in front of the 5′ of hACE2 CDS. Genes are not drawn to scale.

Investigators at the Institute for Antiviral Research at Utah State University have participated in the Collaborative Antiviral Testing Group (CATG) of the National Institutes of Health (NIH) for more than 25 years. As part of the CATG, our research is focused on developing small animal models for emerging viruses to evaluate potential antiviral therapeutics. NIH funding for this project included generation of transgenic hACE2-hamsters to model the viral respiratory infection observed in severe human infections with COVID-19, and then to validate that model for use in evaluation of medical countermeasures. Briefly, we initially generated seven independent founder lines (F0) of transgenic hACE2-hamsters. The F0 animals of each line were then crossbred with wild-type golden Syrian hamsters to produce a heterozygous F1 generation. For each founder line, the F1 animals were crossbred to create heterozygous F2 hamsters that were used in the majority of experimental studies. SARS-CoV-2 infection studies for all three generations (F0, F1, and F2) of each founder line were completed as animals became available. Therefore, differences in virus susceptibility among founder lines were not identified until enough studies were completed to compare the different lines statistically.

In order to demonstrate the utility of this COVID-19 infection model for screening the efficacy of medical countermeasures against COVID-19, we needed a positive control for use in evaluation of potential antivirals. We determined the protective effect of the Toll-Like Receptor 3 (TLR3) agonist polyinosinic-polycytidylic acid (Poly (I:C)) against a SARS-CoV-2 infection in hACE2-hamsters. Poly (I:C) serves as a strong inducer of a type I IFN response via TLR3 signaling and has demonstrated efficacy as a treatment for a SARS-CoV-1 infection in BALB/c mice [18]. Treatment with Poly (I:C) and related compounds results in an increased antiviral response in the host, which limits viral infection. The efficiency of type I IFN induction shown by Poly (I:C) is well established against a variety of viral infections and has been proposed as a prophylactic treatment in the case of a pandemic [19]. The strategy of inducing a strong type I IFN response to overcome SARS-CoV-2 infections has also been evaluated in human patients with the use of pegylated-IFN-2b [20]. However, immune evasion strategies exhibited by SARS-CoV-2 have been shown to suppress the early IFN type I response [21]. Treatment with a compound that stimulates a type I IFN response could mitigate the ability of SARS-CoV-2 to suppress the type I IFN response. We observed that treatment with Poly (I:C) administered prophylactically (before virus infection) decreased morbidity, reduced clinical signs, and significantly improved survival in hamsters. However, the acute nature of the SARS-CoV-2 infection in hACE2-hamsters made treatment therapeutically (after virus infection) more difficult.

In summary, we successfully developed a lethal model for COVID-19 in transgenic hACE2-hamsters. The model included both male and female hACE2-hamsters at 8 weeks of age challenged with the ancestral SARS-CoV-2 (USA_WA1/2020) by the intranasal route, using a 0.1 mL volume containing 10^0.3^ CCID_50_ of infectious virus. While evaluating different treatment regimens using the TLR3 agonist Poly (I:C), we observed higher than expected variability in lethality following infection of the transgenic hamsters. To validate the model, we sought to determine the cause of variability and observed that susceptibility to SARS-CoV-2 infection was dependent on the transgenic hamster founder line (two highly susceptible and two less susceptible), and that prophylactic but not therapeutic treatment with Poly (I:C) was protective in this COVID-19 model of highly susceptible hACE2-hamsters.

## 2. Materials and Methods

### 2.1. Construction of a Gateway Expression Vector pmhyGNEIE-3-K18-hACE2

The plasmid pmhyGNEIE-3-K18-hACE was constructed using the Gateway cloning system (Invitrogen, Carlsbad, CA, USA) per the manufacturer’s instructions. Briefly, the purified 6.6 kb DNA fragment generated from an HpaI and XbaI double digest of pK18-hACE2 plasmid [22] was ligated with the backbone of pENTRTM-1A, an enter vector, which was double digested with Xmn I + Xba I to generate the donor vector of pENTRTM-1A-K18-hACE2. Then, the donor vector was recombined with pmhyGENIE-3 by LR clonase enzyme to generate the expression vector pmhyGNEIE-3-K18-hACE2. Sequencing was performed to identify the pmhyGNEIE-3-K18-hACE2 expression vector before pronuclear (PN) injection.

### 2.2. Isolation of Hamster Zygotes

Female golden Syrian hamsters were induced to superovulate by intraperitoneal (IP) injection of 10–25 IU of pregnant mare serum gonadotropin (PMSG) (BioVendor Cat: RP1782725000) based on body weight in the morning (9–12 a.m.) on Day 1 of the estrous cycle. These females were mated to fertile males at 7:00 p.m. on Day 4 of the estrous cycle and were euthanized approximately 18 h after mating for zygotes isolation. The zygotes were flushed from oviducts with warmed and equilibrated embryo culture *medium*-9 HECM-9 medium (Hyclone Labs, Logan, UT, USA) supplemented with 0.5 mg/mL human serum albumin. The embryos were then washed twice with HECM-9, transferred into 20 µL drops of HECM-9, covered by mineral oil, and cultured at 37.5 °C under 10% CO_2_, 5% O_2_, and 85% N_2_.

### 2.3. Pronuclear Injection of pmhyGNEIE-3-K18-hACE2 Plasmid and Embryo Transfer

The pK18-hACE2 plasmid was a gift from Drs. Paul McCray and Stanley Perlman (University of Iowa, Iowa, IA, USA) [22]. The plasmid was diluted to 6 ng/uL using pH 7.0 TE buffer. The experiments were performed in a dark room, and red filters were used for all microscope light sources. To perform PN injections, a group of 15–20 hamster zygotes were transferred to a 100 µL HECM-9 drop in a microinjection dish, and plasmid was injected into the swollen male pronucleus. After injection, embryos were washed twice with equilibrated HECM-9 and cultured at 37.5 °C under 10% CO_2_, 5% O_2_, and 85% N_2_ before embryo transfer. Embryos with normal morphology were selected to transfer into the oviducts of pseudo-pregnant recipients that were prepared by mating with vasectomized males one day previously (at the same time when zygote donors were mated). Embryos were transferred bilaterally with 10–15 embryos per oviduct. Pups were genotyped at the age of 2 weeks.

### 2.4. PCR Analysis of Transgene in hACE2-Hamster Pups

Genomic DNA was isolated from toe clippings using a Qiagen Blood and Tissue Kit (Qiagen Cat: 69506). Hamsters transgenic for hACE2 expression were detected by PCR analysis using forward primer ACCTGGCTGAAAGACCAGAACAAG and reverse primer AATTAGCCACTCGCACATCC [22]. PCR was performed with the Ex Taq (Takara, Cat: RR001A) and the following parameters: initial denaturation at 94 °C for 5 min followed by 32 cycles of 30 s denaturation at 94 °C, 30 s annealing at 58 °C, and 3 min extension at 72 °C, with a final extension step of 72 °C for 10 min.

### 2.5. Animal Studies

Eight-week-old male and female transgenic hACE2-hamsters were obtained from a specific-pathogen-free colony maintained at the Laboratory Animal Research Center (LARC) of Utah State University (Logan, UT, USA). The hamsters were maintained on Teklad Rodent Diet (Harlan Teklad, Indianapolis, IN, USA) and tap water. Following generation and genotyping of hACE2-pups, hamsters were bred and offspring were used in animal studies. Male and female hACE2-hamsters were randomized into groups and allowed to acclimatize to the BSL-3 facility for 5–7 days prior to the experiment. Animal experiments were completed in the LARC BSL-3 facility under an approved IACUC protocol and with compliance to all ethical regulations.

### 2.6. SARS-CoV-2 Challenge Studies

Severe acute respiratory syndrome associated coronavirus 2 (SARS-CoV-2, USA_WA1/2020 strain) was obtained from the World Reference Center for Emerging Viruses and Arboviruses (WRCEVA, Galveston, TX, USA). After receipt, a virus challenge stock was produced by three passages in Vero 76 cells (American Type Culture Collection, Manassas, VA (ATCC, Manassas, VA, USA). All procedures were performed inside a biosafety cabinet in a BSL-3 facility, and all personnel wore controlled air-purifying respirators (MaxAir systems, Irvine, CA, USA). For viral challenge, hamsters were anesthetized via intraperitoneal (IP) injection with ketamine (150 mg/kg) and xylazine (10 mg/kg), followed by intra-nasal (IN) inoculation of virus challenge (1 × 10^0.3^ CCID_50_) in 100 µL of MEM. Infected hamsters were monitored twice daily for clinical signs of infection, weight loss, and mortality. Sample collection for virus titer determination and histopathology included euthanasia of hamsters on days 2-, 4-, 6-, and 8-p.i. by IP injection of pentobarbital sodium and phenytoin sodium (200 mg/kg euthanasia solution, Vet One^®^, Boise, ID, USA). Tissue homogenates for viral load determination were obtained by homogenizing tissues in MEM and storage at −80 °C. Oropharyngeal swabs were collected from hamster’s oropharynx daily for the first 5 days of infection with sterile nylon flocked swabs in MEM.

### 2.7. Clinical Signs for Disease Scoring

Hamsters were observed twice daily throughout the course of the study for clinical signs following SARS-CoV-2 infection. The clinical signs observed for each hamster were given a numerical value based on the perceived severity of the sign. Numerical scores for clinical signs were as follows: ruffled fur (1), hunched position (1), nasal and ocular discharge (1), diarrhea (1), abnormal gait (2), lethargy (2), mild to moderate dyspnea (3–5), severe dyspnea (7), and abdominal swelling (9). The daily clinical score was the summation of all observed signs for each hamster. Hamsters were euthanized if they showed ≥20% weight loss, or if the total clinical score on any single day was ≥9. For euthanasia criteria based on observation of clinical scores, see Table S1: Scores for Clinical Signs Observed in SARS-CoV-2 Infected hACE2-Hamsters (Supplementary Information).

### 2.8. Quantification of Virus in Hamster Tissue

Tissue viral titers were determined via endpoint dilution assay. Briefly, homogenized tissue samples were serially diluted (10-fold) and plated into 4 replicate wells on 96-well plates containing Vero 76 cell monolayers seeded 24 h prior to sample titration. The plates were incubated at 37 °C with 5% CO_2_ and then scored by visual observation on day 6 under a light microscope for the presence of cytopathic effects (CPE). Fifty percent cell culture infectious doses (CCID_50_) per gram of sample were calculated using the Reed–Muench method [23] prior to statistical analysis.

### 2.9. Poly (I:C)] Treatment

Poly (I:C), a Toll-Like Receptor 3 (TLR3) agonist, was evaluated as a positive control for virus challenge dose in potential antiviral drug studies. Infected hamsters were treated twice daily for 2 days, beginning 24 h prior to virus challenge, via IP injection with 10 mg/kg/d Poly (I:C) 10 (InvivoGen, San Diego, CA, USA).

### 2.10. Histopathological Analyses

Tissue samples, including nasal turbinate, trachea, lung, heart, liver, kidney, adrenal gland, spleen, stomach, intestinal tract, pancreas, testes, epididymis, ovary, uterus, skeletal muscle, bone marrow, eye, and brain, were preserved in 10% neutral buffered formalin for 48 h and then moved to 70% ethanol until trimming and embedding. Fixed tissue sections were processed and embedded in paraffin according to routine histologic techniques. Sections 5 µm thick were stained with hematoxylin and eosin (H&E) and examined via light microscopy by a board-certified veterinary pathologist. In tissues where lesions were present, the severity was scored subjectively on a scale of 0–4, with 0 indicating no lesions and 4 indicating severe lesions.

### 2.11. Ethical Treatment of Animals

This study was completed under the approval of the Institutional Animal Care and Use Committee of Utah State University (Logan, UT, USA). The procedures were performed in the AAALAC-accredited Laboratory Animal Research Center of Utah State University (Logan, UT, USA) in accordance to the National Institutes of Health Guide for the Care and Use of Laboratory Animals [24].

### 2.12. Statistical Analyses

Kaplan–Meier survival curves were generated and compared by the Log-rank (Mantel–Cox) test followed by pairwise comparison using the Gehan–Breslow–Wilcoxon test in GraphPad Prism 10.2.1 (GraphPad Software, San Diego, CA, USA). The mean body weights were analyzed using analysis of variance (ANOVA) followed by Tukey’s multiple comparison test using Prism 10.2.1. Virus titer differences were evaluated using ANOVA on log-transformed values, assuming equal variance and normal distribution. Following ANOVA, individual treatment values were compared to placebo controls via Tukey’s pair-wise comparison test using Prism 10.2.1. Clinical scores were compared via non-parametric Kruskal–Wallis test using Prism 10.2.1.

## 3. Results

### 3.1. Virus Challenge of hACE2-Hamster Founder Lines

Following generation of the transgenic hamsters, we produced a total of 53 F0 pups, out of which 22 were identified as carrying the hACE2 transgene. Before establishing breeding colonies from these F0 founder hACE2-hamsters, we randomly selected animals from different founders and compared their susceptibility to SARS-CoV-2 infection and associated pathogenesis. Hamsters were challenged intranasally (IN) with an infectious dose comparable to the infectious dose used in the wild-type hamster model (1 × 10^4.3^ CCID_50_) [14]. SARS-CoV-2 challenge of the F0 generation hamsters resulted in partial mortality, 50–70%. Based on these encouraging results, we bred the remaining seven F0 founder hamsters and established independent breeding colonies from each of the founders.

Critical evaluation of the founder lines included a series of challenge dose titrations that identified the optimum challenge dose, time of virus shedding, replication in different tissues, and identification of a treatment for use as a positive control in evaluating potential medical countermeasures (Supplementary Information Tables S1 and S2). In addition, hACE2 expression levels in transgenic animals were evaluated via RT-PCR and co-localization of human and hamster ACE2 by immunofluorescence (IF) (Supplementary Information Figures S1–S3), although further characterization was beyond the scope of our funding.

**Figure 2:**
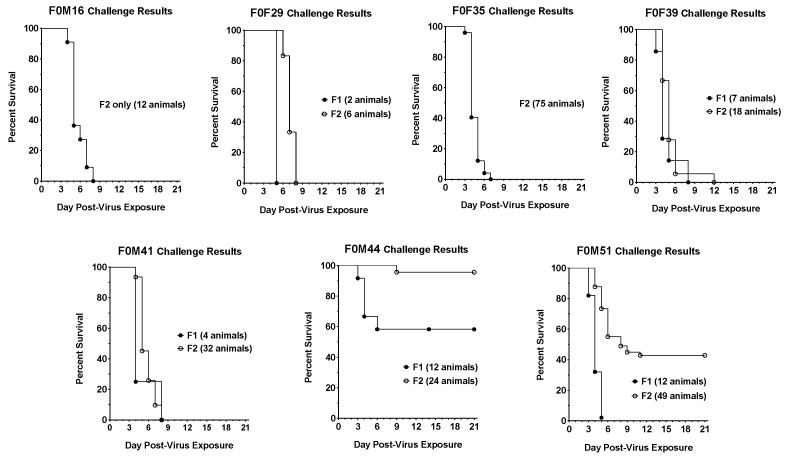
Survival curves for F1 and F2 generations of hACE2-hamster founder lines infected with SARS-CoV-2. In five of the founder lines, F0M16, F0M29, F0M35, F0M39, and F0M41, the susceptibility to a lethal SARS-CoV-2 infectious dose of 10^4.3^ CCID_50_ appears similar between the F1 and F2 generations. In two founder lines, F0M44 and F0M51, the susceptibility to the virus decreases between the F1 and F2 generations. The varying susceptibilities could be a result of founders exhibiting a mosaic expression of the hACE2 transgene in different founder lines.

Infection of the F1 and F2 generations demonstrated that hamsters from different founder lines varied in their susceptibility to SARS-CoV-2 challenge (Figure 2). Two lines (F0M44 and F0M51) were designated less susceptible to lethal infection because viral challenge resulted in lower mortality (<60%). Five founder lines (F0M16, F0M29, F0M35, F0M39, and F0M41) were designated as highly susceptible to SARS-CoV-2 challenge because infection resulted in 90–100% mortality. There could be several reasons for the different levels of virus susceptibility observed among transgenic hamster lines. First, given the nature of piggyBac-mediated transgenesis, where a transgene can be integrated into any TTAA sites in the animal genome, different expression levels of the transgene may occur. Indeed, recent analyses using targeted locus amplification [24] to map the hACE2 transgene integration site(s) in the seven F0 hamster founders showed that the hACE2 transgene was integrated into different genomic loci among each of the F0 founder hamsters. Interestingly, except for founder F0M51, which carries two copies of the hACE2 transgene, one on an autosome and the other on the X-chromosome, each of the other six founder hamsters carries a single copy of the hACE2 transgene. Second, because the Syrian hamster is considered outbred, each of the transgenic lines reported here was established from a different F0 founder hamster. It is possible that genetic variations among the transgenic lines also contribute to the different susceptibilities. This second possibility could be the most likely reason for the different susceptibilities between F1 and F2 hamsters observed within the F0M44 and F0M51, respectively (Figure 2).

**Figure 3:**
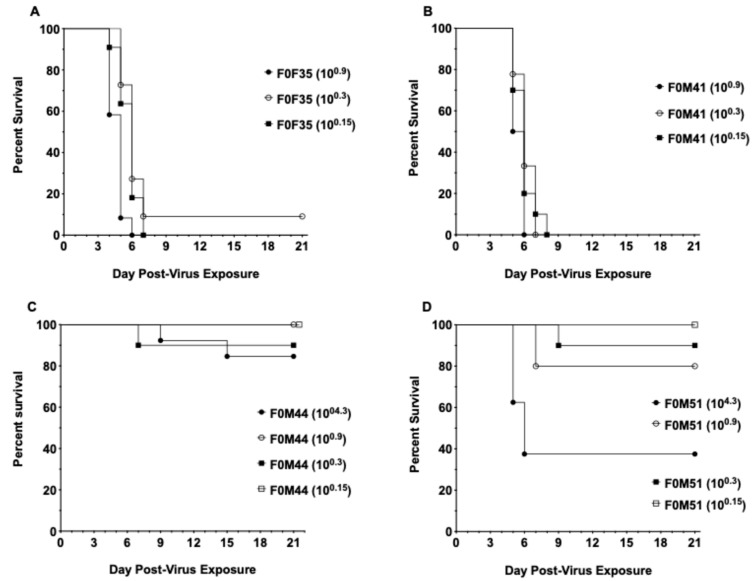
Survival curves for hACE2-hamster founder lines differing in susceptibility to SARS-CoV-2. Groups of 8-week-old hACE2-hamsters (*n* = 10/virus dose) infected with SARS-CoV-2. (**A**) Viral challenge in F0F35 ^highly susc^ line resulted in 90–100% mortality (**B**) and 100% mortality in F0M41^highly susc^ line. (**C**) Challenge in the F0M44^less susc^ line resulted in 0–10% mortality. (**D**) Infection in the F0M51^less susc^ line resulted in 0–20% mortality when challenged with a series of low infectious doses and 60% mortality with a high dose.

Two of the founder lines that were highly susceptible to lethal disease (F0M35^highly susc^ and F0M41^highly susc^) and two of the lines that were less susceptible to lethal disease (F0M44^less susc^ and F0M51^less susc^) were selected for future infection studies. These four founder lines were compared in a series of challenge dose titrations to confirm the varying susceptibility to SARS-CoV-2 infection (Figure 3). Infection of animals from the F0M35^highly susc^ and F0M41^highly susc^ founder lines resulted in 90–100% mortality at all three low infectious doses of 10^0.9^, 10^0.3^, and 10^0.15^ CCID_50_. In animals from the F0M51^less susc^ founder line, low IDs resulted in 0–20% mortality, and a high infectious dose (10^4.3^ CCID_50_) produced 60% mortality. Infection of animals from the F0M44^less susc^ line resulted in no mortality at 10^0.9^ and 10^0.15^ CCID_50_, 10% mortality at 10^0.3^ CCID_50_, and 20% at 10^4.3^ CCID_50_.

### 3.2. Clinical Signs Observed in hACE2-Hamsters After Virus Challenge

**Figure 4:**
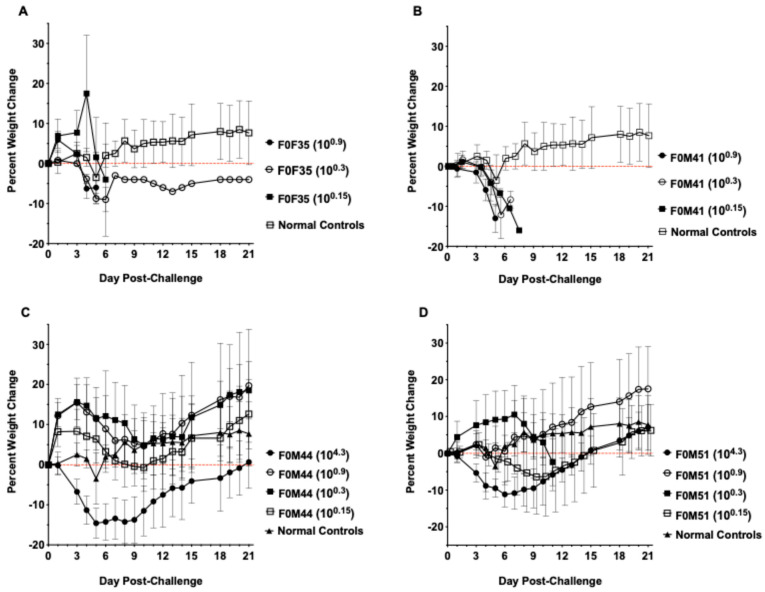
Percent weight loss in hACE2-hamster founder lines differing in susceptibility to SARS-CoV-2. Groups of 8-week-old hACE2-hamsters (*n* = 10/virus dose) infected with SARS-CoV-2. (**A**,**B**) Hamsters from the F035^highly susc^ and F041^highly susc^ lines lost significant weight prior to mortality when challenged with a low dose (10^0.3^ CCID_50_). (**C**,**D**) In the F0M44 ^highly susc^ and F0M51 ^highly susc^ lines, hamsters experienced significant weight loss only when challenged with the high dose (10^4.3^ CCID_50_).

**Figure 5:**
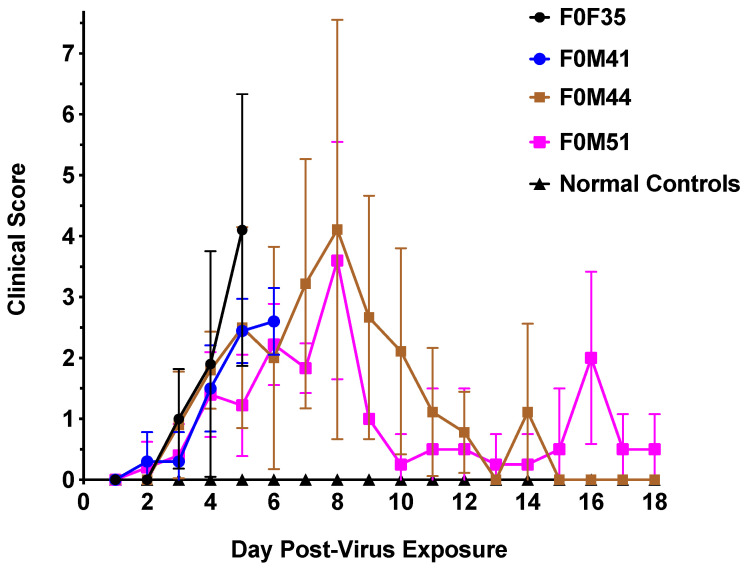
Clinical scores in hACE2-hamster founder lines differing in susceptibility to SARS-CoV-2. Groups of 8-week-old hACE2-hamsters (n = 10) infected with SARS-CoV-2. Hamsters in the F0M35^highly susc^ and F0M41^highly susc^ groups were infected with an infectious dose of 10^0.3^ CCID_50_, whereas the animals in the F0M44^less susc^ and F0M51^less susc^ were infected with an infectious dose of 10^4.3^ CCID_50_. The clinical scores for all 4 founder lines began to increase at day 2 p.i. and continued to rise until either mortality in F0M35^highly susc^ and F0M41^highly susc^ or mean peak scores of 4.1 and 3.6 at day 8 p.i. in the F0M44^less susc^ and F0M51^less susc^ lines, respectively. Following day 8, the F0M44^less susc^ and F0M51^less susc^ hamsters began to recover from infection at which point clinical scores decreased.

Although infection in transgenic hamster lines that were less susceptible to lethal disease resulted in increased survival, similar signs of morbidity such as weight loss (Figure 4) and clinical signs of infection (Figure 5) were observed in all founder lines following viral challenge. Hamsters from the F035^highly susc^ and F041^highly susc^ lines began to lose weight starting day 3 p.i., losing an average of 10–15% of body weight prior to death regardless of infectious dose. The low dose (10^0.3^ CCID_50_) virus challenge resulted in limited weight loss in F0M44^less susc^ and F0M51^less susc^ animals, whereas a high dose (10^4.3^ CCID_50_) resulted in weight loss comparable to the highly susceptible lines. All hamsters were evaluated twice daily for clinical signs of SARS-CoV-2 infection. Clinical signs observed in hamsters included general signs of morbidity in rodents such as ruffled fur, abnormal gait, and hunched position. Other clinical signs of viral infection were observed including lethargy, nasal/ocular discharge, diarrhea, and moderate to severe dyspnea. SARS-CoV-2 infection of F0M35^highly susc^ and F0M41^highly susc^ hamsters resulted in clinical signs that increased in severity as the infection progressed through day 6 p.i.. Severe signs included respiratory distress and mortality. In a limited number of infected animals (<2%), the intestines and colon were severely distended as observed upon necropsy on day 4 p.i.. In the F0M44^less susc^ and F0M51^less susc^ lines, clinical signs peaked on day 8 p.i. after which they began to decrease as the hamsters recovered from the infection.

### 3.3. Virus Titers in Hamster Tissue

**Figure 6:**
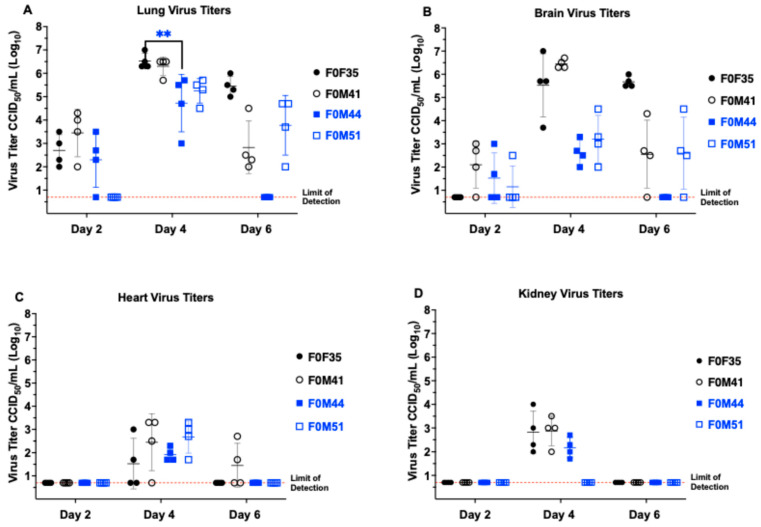
Virus tissue titers in hACE2-hamster founder lines differing in susceptibility to SARS-CoV-2. Groups of 8-week-old hACE2-hamsters infected with SARS-CoV-2 were sacrificed on days 2, 4, and 6 post-infection (*n* = 4/day). Titers for F0F35^highly susc^ and F0M41^highly susc^, shown in black, are after a 10^0.3^ CCID_50_ virus challenge dose. Titers for F0F35^highly susc^ and F0M41^highly susc^ animals, shown in black, are after a 10^0.3^ CCID_50_ virus challenge dose. Titers for F0M44^less susc^ and F0M51^less susc^ animals, shown in blue, are after a 10^4.3^ CCID_50_ challenge dose. (**A**) SARS-CoV-2 is first detectable at day 2 p.i. and increases to a peak in lung tissue at day 4 p.i., after which it decreases by day 6 p.i.. (**B**) Virus titer reaches a peak in brain tissue at day 4 p.i. and, like lung tissue, begins to decrease by day 6 p.i.. (**C**) Infectious virus is detectable in the cardiac tissue at day 4 p.i. for all 4 founder lines and at day 6 p.i. in 1 of F0M41^highly susc^ animal. (**D**) Virus was only detected in renal tissue on day 4 p.i. in 3 of the 4 founder lines (F0F35, F0M41, and F0F44). The only significant difference observed between founder lines post-infection was in lungs on day 4 p.i. (** *p* < 0.01). In addition, hACE2 mRNA expression is high in in all tissues in which virus replication was observed (See Figure S1).

Hamsters from different founder lines were sacrificed on days 2, 4, and 6 p.i. to evaluate tissue virus titers (Figure 6). SARS-CoV-2 was detectable in both lung and brain tissues from hACE2-hamsters beginning day 2 p.i. Interestingly, when animals were challenged with a low dose (10^0.3^ CCID_50_), the viral titers in both lung and brain tissues were comparable at day 2 p.i.. In contrast, when challenged with a high dose (10^4.3^ CCID_50_), the amount of virus found in lung tissue at day 2 p.i. was ~3 logs higher than brain tissue. Regardless of the challenge dose, titers in both these tissues increased to a peak at day 4 p.i. and declined by day 6 p.i. We suspect that the early decrease in virus titers in the lung on day 6 is due to the tissue damage and severe inflammatory response targeting and eliminating the virus. In the wild-type hamster model, detection of virus peaks at day 3 to 4 post-infection then decreases and disappears between day 6 to 10 p.i. due to virus clearance. In addition, peak lung and brain virus titer were temporally associated with detectable SARS-CoV-2 in cardiac and renal tissues, plus low levels of virus in olfactory bulbs. hACE2 mRNA expression analyses showed that hACE2 expression is observed in lung, brain, heart, kidney, liver, spleen, and small intestinal tissues (Figure S1). However, in the limited number of animals evaluated by RT-PCR, significant differences between founder lines were not observed.

### 3.4. Histopathology in hACE2-Hamsters Infected with SARS-CoV-2

**Figure 7:**
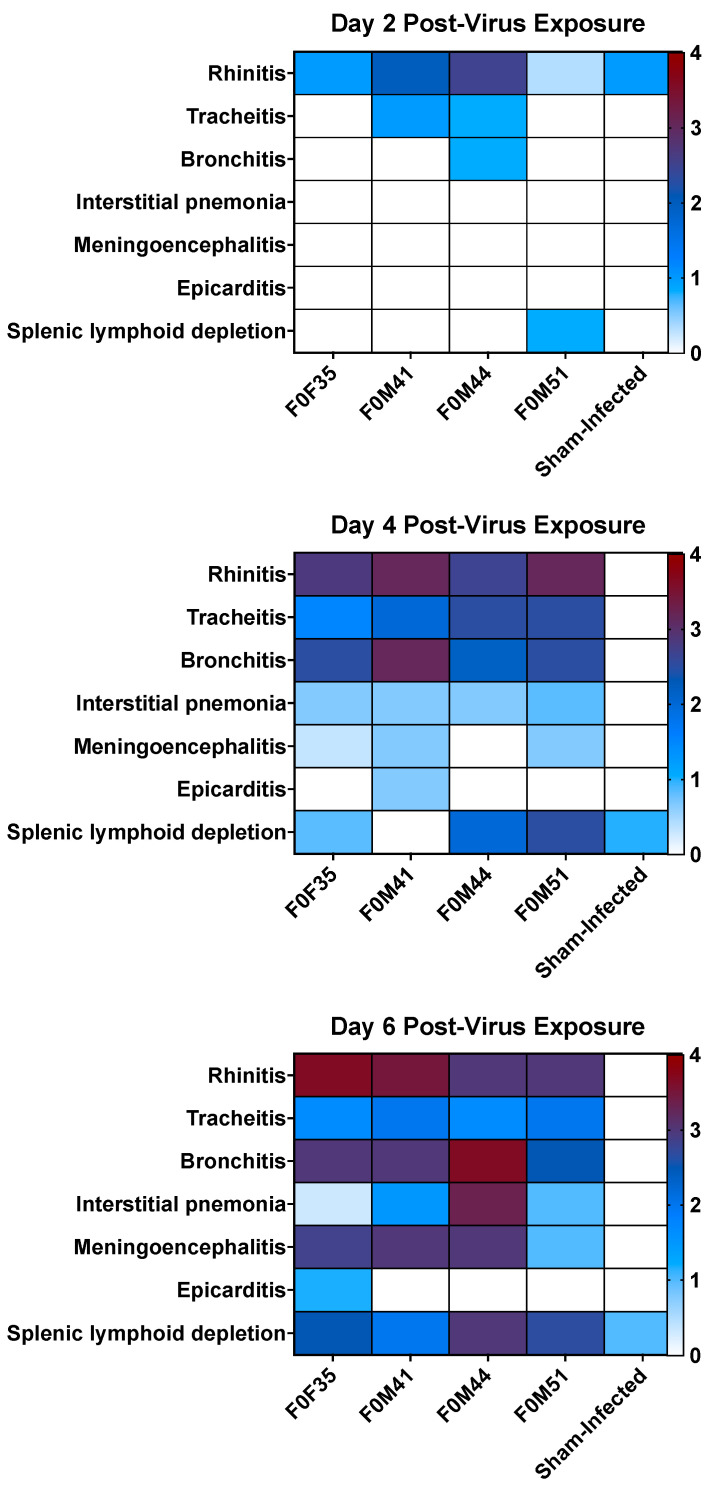
Summary of histological lesions in ACE2-hamster founder lines differing in susceptibility to SARS-CoV-2. Groups of 8-week-old hACE2-hamsters (*n* = 3/day) infected with 10^0^^.3^ CCID_50_ SARS-CoV-2. Hamsters from the F0M44^less susc^ line had the most respiratory lesions at day 2 p.i. and developed a moderate to severe infection of both upper and lower respiratory tracts. In addition, animals from the F0M35^highly susc^, F0M41^highly susc^, and F0M44^less susc^ lines developed moderate meningoencephalitis. However, over the course of infection, the F0M51^less susc^ line developed the lowest lesion scores.

Figure 7 shows a summary of histological lesions in hACE2-hamster founder lines after SARS-CoV-2 infection. Lesions of neutrophilic rhinitis with epithelial necrosis were observed in 5 out of 12 total founders (three hamsters from each line sacrificed per day) with the F0M44^less susc^ line showing the highest clinical signs on at day 2 p.i.. The rhinitis lesions progressed to a neutrophilic and lymphoplasmacytic rhinitis with variable respiratory epithelial cell necrosis, restitution, and regeneration in all hamsters at days 4, 6, and 8 p.i.. Tracheitis was present in two out of nine hamsters at day 2 p.i. and in all hamsters at days 4, 6, and 8 p.i.. Tracheal lesions were characterized by neutrophilic and lymphoplasmacytic infiltration in the lamina propria with accumulation of neutrophils in the lumen and variable epithelial cell necrosis, attenuation, and regeneration. Bronchitis was present in 1 out of 12 hamsters at day 2 p.i., then bronchitis and bronchiolitis or bronchointerstitial pneumonia was observed in 12 out of 12 hamsters on day 4 p.i.. The lesions of bronchitis and bronchiolitis were characterized by epithelial necrosis and infiltration of neutrophils that migrated towards the epithelium and accumulated in the lumen.

**Figure 8:**
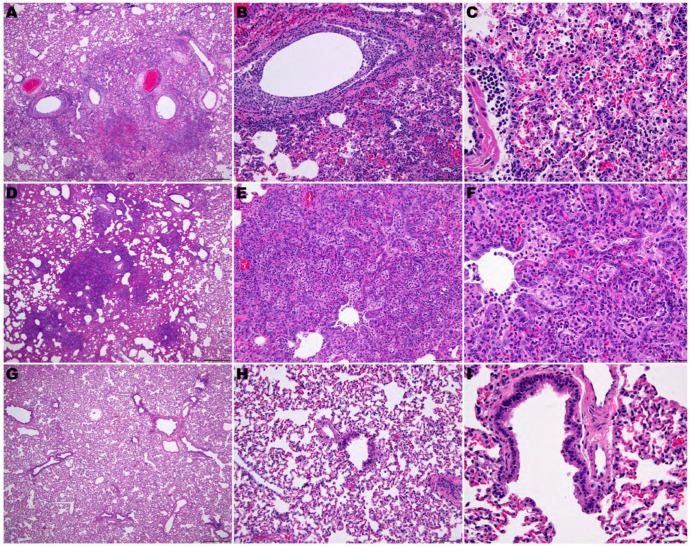
Pulmonary pathology of SARS-CoV-2-infected F035^Highly Susc^ hACE2-hamsters. (**A**–**C**) SARS-CoV-2 (10^0.3^ CCID_50_)-infected hamster at day 6 p.i.. (**D**–**F**) SARS-CoV-2-infected hamster at day 8 p.i.. (**G**–**I**) Sham-infected hamster. (**A**) Inflammatory reaction and necrosis centered on bronchi and bronchiole extending into adjacent alveoli. (**B**) Bronchiolar epithelial cell necrosis with neutrophilic and lymphocytic inflammation. Alveolar septae are thickened by inflammatory cells. (**C**) Edema fluid, fibrin, neutrophils, and macrophages fill alveoli and expend alveolar septa. (**D**) Early resolution of bronchointerstitial pneumonia. Alveolar septae are prominent around bronchioles. (**E**,**F**) Alveolar septa are prominent due to marked pneumocytes type Il hyperplasia. Alveolar spaces are filled with edema fluid, fibrin, neutrophils, and macrophages. (**G**–**I**) Lung of a sham-infected hamster. (**H**,**E**) staining. (**A**,**D**,**E**): 40×. Bar = 500 μm. (**B**,**E**,**H**): 200×. Bar = 100 μm. (**C**,**F**,**I**): 400×.

**Figure 9:**
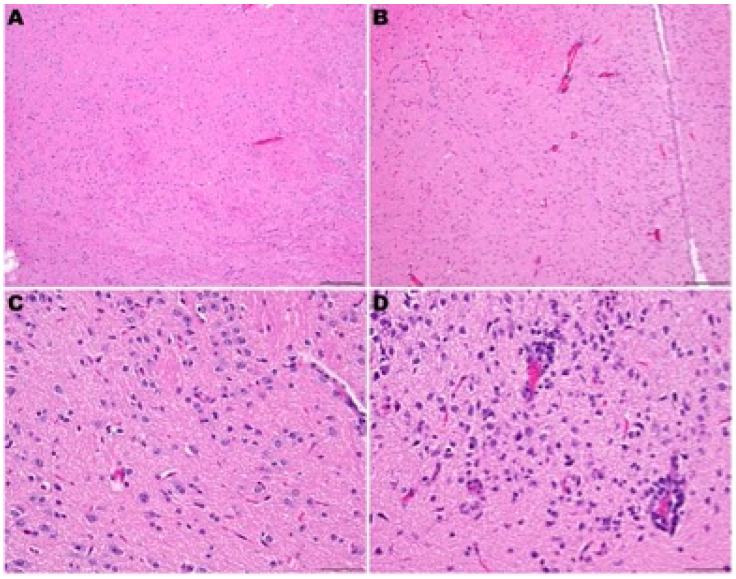
Neuropathology of SARS-CoV-2-infected F035^Highly Susc^ hACE2-hamsters. (**A**,**C**) Sham-infected hamster at day 6 p.i.. (**B**,**D**) SARS-CoV-2 (10^0.3^ CCID_50_)-infected hamster. Figure (**B**): Mild lymphocytic perivascular cuffing in the thalamus. Figure (**D**): Gliosis and lymphocytic perivascular cuffing in the thalamus. H&E staining. (**A**,**C**): 100×. Bar = 200 μm. (**C**,**D**): 400×.

Macrophages, lymphocytes, and plasma cells also infiltrated the lamina propria. In addition, infiltration of alveolar septae surrounding terminal bronchioles by macrophages, neutrophils, lymphocytes, and plasma cells preceded mortality or euthanasia on days 5–7 p.i.. Perivascular infiltration of lymphocytes sometimes with edema was observed multifocally around larger caliber blood vessels. In addition, edema fluid with fibrin and variable numbers of macrophages and neutrophils were in alveolar spaces. At day 8 p.i., evidence of early resolution was observed with minimal lesions present in the bronchi, prominent pneumocyte type II hyperplasia was observed in alveoli and alveolar spaces containing macrophages and few to moderate numbers of neutrophils with occasional syncytial cells (Figure 8). In addition to lung lesions, at day 4 p.i., lymphocytic encephalitis or meningoencephalitis was observed in 6 out of 10 hamsters. By day 6 p.i., 8 out of 10 hamsters had developed encephalitis. In addition, both hamsters that were alive at day 8 p.i. had encephalitis. Lymphocytic perivascular cuffing within the brain and meninges and presumptive mild gliosis were most frequently observed in the brain stem and thalamus (Figure 9). In addition, lesions in heart tissues have been described in human SARS-CoV-2 infections and were observed in several hACE2-hamsters, but these lesions were not observed consistently [25,26,27,28]. No other microscopic changes were detected in sections of liver, kidney, adrenal gland, stomach, intestinal tract, pancreas, testes, epididymis, ovary, uterus, skeletal muscle, bone marrow, or eye.

Comparison of the pathology observed in the four founder lines demonstrated that a low dose (10^0.3^ CCID_50_) resulted in lesions in the upper respiratory tract, with mild lower tract involvement in hamsters from the F0M51^less susc^ line (Figure 9). However, infection of animals from the F0M44^less susc^ line resulted in a severe infection in both the upper and lower respiratory tract and moderate encephalitis. Infection of hamsters from the F0M35^highly susc^ and F0M41^highly susc^ lines resulted in moderate to severe lesions observed in brain tissue by day 6 p.i.. The lesions observed in lung tissue were most severe at day 4 p.i. and began to resolve by day 6 p.i.. In one animal from the F0M44^less susc^ line, lesions in the brain tissue were comparable to brain tissue observed in infected hamsters from the highly susceptible lines. However, hamsters from this line developed severe pathology in the lower respiratory tract on day 6 p.i. (Figure 9). Despite the observation of more severe respiratory disease lesions in offspring from the F0M44^less susc^ founder line, these animals were less susceptible to lethal disease. In previous studies of SARS-CoV-2 infection in hACE2-hamsters, the major pathological findings consisted of multifocal gliosis throughout the gray matter, meningitis, perivascular inflammation, and neuronal necrosis, including pathology in the brain stem (medulla) [28]. Taken together, these findings suggest that mortality in the infected hamsters was most likely due to the neurologic damage observed in the founder lines that are highly susceptible to lethal disease.

### 3.5. Poly (I:C) Treatment for a SARS-CoV-2 Infection in hACE2-Hamsters

A positive control was necessary to establish an infection model to evaluate potential antiviral therapeutics. Poly (I:C) is a synthetic analog of double-stranded RNA. As an agonist for Toll-Like Receptor 3 (TLR3), Poly (I:C) functions as an immunostimulant, activating the transcription factor interferon regulatory factor 3 (IRF3) to produce type I IFNs (e.g., IFN-β) [18,29].

**Figure 10:**
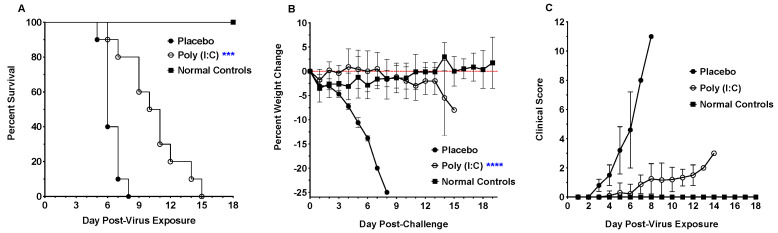
Effect of prophylactic treatment with Poly (I:C) on SARS-CoV-2 morbidity and mortality in F041^Highly Susc^ hACE2-hamsters. Groups of 8-week-old hACE2-hamsters (*n* = 10) infected with 10^0.3^ CCID_50_ SARS-CoV-2. (**A**) Poly (I:C) treatment significantly increased the MDD for treated hamsters. (**B**) Treatment with Poly(I:C) prevented weight loss over the course of the infection. (**C**) Clinical scores of treated hamsters decreased compared to placebo controls. Daily clinical signs and scores following infection consisted of rough coat (1), nasal or ocular discharge (1), hunched posture (1), abnormal gait (2), lethargy (2), and mild to moderate dyspnea (1 to 3), with each animal scored daily. (*** *p* < 0.01, **** *p* < 0.0001).

**Figure 11:**
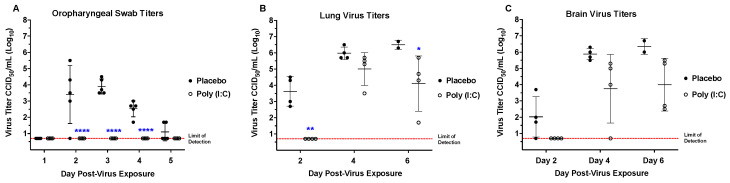
Prophylactic treatment with Poly(I:C) prevents viral shedding and reduces lung virus titers in F041^Highly Susc^ hACE2-hamsters. Groups of 8-week-old hACE2-hamsters (*n* = 5/day) infected with 10^0.3^ CCID_50_ SARS-CoV-2. (**A**) Infectious virus was not observed in oropharyngeal swabs from treated animals, in contrast to placebo-treated hamsters. (**B**) Virus titer was significantly reduced in the lung tissue from treated hamsters on days 2 and 6 p.i.. (**C**) Poly (I:C) treatment reduced virus titers in the brain, but the reduction was not significant. (* *p* < 0.05, ** *p* < 0.1, **** *p* < 0.0001).

Prophylactic treatment with Poly (I:C) significantly prolonged the mean day of death (MDD) as treated hamsters survived 5–7 days longer than placebo-treated hamsters, after which treated animals began to succumb to infection (Figure 10). Along with an increase in the MDD, treatment also decreased morbidity in hamsters as evidenced by protection from weight loss and reduced clinical signs. Infectious virus was not observed in oropharyngeal swabs from treated animals, in contrast to placebos (Figure 11). In addition, lung virus titers in hamsters treated with Poly (I:C) were significantly reduced at days 2 and 6 p.i. (Figure 11). However, brain virus titers decreased but the reduction was not statistically significant (Figure 11). Surprisingly, treatment with Poly (I:C) beginning 4 h post-infection or additional dosing after prophylactic treatment did not provide a survival benefit.

## 4. Discussion

SARS-CoV-2 challenge in hACE2-hamsters resulted in weight loss greater than 15%, clinical signs of infection, and mortality. Consistent with current research in Syrian hamster models, infectious virus was first detectable via oropharyngeal swabs between 24 to 48 h post-infection, and the virus replicated to detectable levels in lung and brain tissue by day 2 p.i. [14,25]. General clinical signs were observable beginning day 2 p.i., comparable to data from previous Syrian hamster models [30].

Progression to more severe clinical signs and death by day 6 p.i. was observed using hACE2-mouse models with viral titers remaining in the brain and renal tissue until death [31]. In the hACE2-hamster model, we also observed that in the days prior to death, the virus titer in both lung and brain tissue increased and virus was detectable in both cardiac and renal tissue. Histopathology revealed that infected hACE2-hamsters developed rhinitis, tracheitis, bronchointerstitial pneumonia, and encephalitis, but did not develop the acute alveolar injury that has been observed in severe COVID-19 human patients [32]. Death in the hACE2-hamster is most likely due to a combination of respiratory and neurologic diseases. However, lesions in the central nervous system are believed to be more significant since similar respiratory tract pathology has been observed in wild-type hamsters without accompanying mortality [30,32]. In human patients, although the primary cause of mortality is considered to be respiratory failure [33], there is also evidence for multi-systemic damage [34]. The hACE2-hamster model of SARS-CoV-2 infection may serve as a valuable model for extra-respiratory pathogenesis, as it recapitulates additional clinical signs besides the respiratory infection observed in wild-type hamsters. To address questions regarding the drivers of lethality and long-term effects of COVID-19 infection, it is crucial to recognize other systems that may be impacted by infection.

Similar differences in susceptibility to infection have been observed in different hACE2-mouse breeder lines [15,16], However, we demonstrated that not all hACE2-hamster founder lines were equally susceptible to lethal challenge with SARS-CoV-2. The F0M44 and F0M51 lines were less susceptible to lethal disease. The F0M51 line is of median susceptibility to lethal disease, with 80–100% mortality achievable when hamsters are challenged with a high dose (10^4.3^ CCID_50_). In contrast, when challenged with the same high virus dose, mortality was only observed in ~10–20% of hamsters from the F0M44 line. There was a noticeable difference between the amount of virus detected in brain tissue from the F0M35^highly susc^ and F0M41^highly susc^ lines compared to the F0M44^less susc^ and F0M51^less susc^ lines at day 4 p.i.. The amount of detectable SARS-CoV-2 in the brain tissue of the highly susceptible lines was ~2 to 3 times higher than in the less susceptible lines, just prior to the onset of severe symptoms. It is also important to note that this peak titer of virus in the brain correlates with the severe respiratory distress observed in hACE2-hamsters. Furthermore, this severe respiratory distress is not witnessed in golden Syrian hamsters [30]. This is interesting since similar pathology is observed in lung tissue from both hamster models, but only the hACE2-hamsters develop signs of severe respiratory distress [30,35]. While lesions observed in the lung tissue from both models are similar, this is not true for lesions observed in brain tissue. Lesions in the CNS have not been observed in wild-type hamsters, whereas severe lesions were observed in hACE2-hamsters [28,31,35]. hACE2mouse and hamster models developed using the K18 promoter develop severe neuropathology following SARS-CoV-2 challenge [36,37,38]. Since severe respiratory pathology has been observed in both wild-type and hACE2-hamsters, we hypothesize that the severe respiratory distress witnessed in hACE2-hamsters highly susceptible to lethal disease may be attributed to the neurologic disease caused by SARS-CoV-2. In addition, low levels of virus were detected in the olfactory bulbs of hamsters, suggesting a possible olfactory route for neuroinvasion.

The histological lesions of bronchitis, interstitial pneumonia, and meningoencephalitis were all significantly different from sham-infected hamsters, although none of these lesions showed significant differences between founder lines. In addition, hACE2 expression levels in tissues from different founder lines was evaluated by RT-PCR and colocalization of human and hamster ACE2 by IF, although significant differences between founder lines was not observed. We suspect genomic analyses will be required to fully elucidate the mechanism for the differences observed in virus susceptibility. However, improvements in the reference genomes for Syrian hamsters are needed before those types of analyses can be completed.

Prophylactic treatment with the TLR3 agonist Poly (I:C), significantly increased the mean day of death in hamsters, while treatment with Poly (I:C) administered after infection (therapeutically) did not protect hamsters from mortality. This is evidenced by the lack of weight loss and clinical scores in treated hamsters over the first 7 to 10 days of infection. After this early protection from infection, treated hamsters succumb to infection within 5–7 days. Animals that survive longer after infection often show more tissue damage. Our veterinary pathologist shared his surprise after observing that wild-type hamsters could survive a respiratory infection resulting in such severe lung damage (A.J.V., personal communication). Considering this observation, one hypothesis is that the unique physiology of Syrian hamsters with the ability to reduce their metabolic activity during torper, similar to hibernation, may provide them with a survival benefit to lung damage [39]. Poly (I:C) treatment significantly reduced viral titers, decreased signs of morbidity, and resulted in a delay in the time course of SARS-CoV-2 infection. Poly (I:C) was originally developed as an anti-cancer therapy that stimulated type 1 interferon production in antigen-presenting cells such as macrophages and dendritic cells [40]. It seems safe to conclude that an initial increase in type 1 interferon production accounts for the lack of morbidity in treated animals early in the course of infection. However, once the initial protective response decreases, the course of infection resumes and the hamsters succumb to infection. We normally prefer a positive control that can be administered therapeutically. However, the acute nature of SARS-CoV-2 infection in highly susceptible hACE2-hamsters has proven difficult to treat after virus challenge. These findings suggest that Poly (I:C) may be a suitable positive control for the SARS-CoV-2 challenge dose in this model to evaluate medical countermeasures.

The hACE2-hamster infection model has the potential to evaluate the drivers of severe COVID-19 and explore neurologic manifestations of infection [9,10,13]. The complex nature of SARS-CoV-2 infections demands that researchers have a variety of tools to study the various disease manifestations and their implications in human cases [4,5,6]. Animal models of infection are among these important tools that allow the study of viral diseases and pathogenesis. Our funding from the National Institute of Health specified an animal model for a viral respiratory infection that progressed to a disease state comparable to that seen in severe human infection with COVID-19. We demonstrated that the hACE2-hamster model provides a sensitive model for a systemic, lethal infection achieved with an exceptionally low 10^0.3^ CCID_50_ challenge dose. However, the acute nature of SARS-CoV-2 infection in the highly susceptible hACE2-hamster founder lines may not provide a treatment window long enough for evaluation of antiviral therapeutics. In addition, these studies demonstrate that hACE2 transgenic hamsters differ in their susceptibility to SARS-CoV-2 infection, based on the transgenic hamster founder line. Evaluation of founder lines with lower susceptibility was beyond the scope of these studies, although modeling different aspects of the complex disease associated with COVID-19 in human patients may be a fruitful area of future research. Furthermore, this model may be useful for evaluating the neuropathology of SARS-CoV-2 infection and its contribution to mortality from COVID-19, as well as development of long-term symptoms (Long COVID-19) [7,8].

During the preparation of this manuscript, the hACE2-hamster model was used by several other research groups whose studies confirmed the lethal phenotypes and reported similar histopathology when the SARS-CoV-2 USA_WA1/2020 strain was used [38]. In addition, the hACE2-hamster model has also been used to study several SARS-CoV-2 variants, including B.1.1.529 [28], BA.2 [41,42], BA.4 and BA.5 [43], BA.2.75 [44], and BA.4.6 and BQ.1.1 [45]. These studies demonstrated that the hACE2-hamsters are susceptible to each of these new SARS-CoV-2 variants and develop similar disease to that caused by the WA1/2020 strain, albeit with much less severity. In addition, we demonstrated the utility of the hACE2-hamster model in testing a human polyclonal IgG immunotherapeutic [46].

## Data Availability

The original contributions presented in the study are included in the article/Supplementary Materials, further inquiries can be directed to the corresponding author.

## Supplementary Materials

The following supporting information can be downloaded at: https://www.mdpi.com/article/10.3390/v16101625/s1, Figure S1: hACE2 expression in tissues from transgenic hamsters by RT-PCR. Figure S2: Co-localization of human and hamster ACE2 in lungs from an infected hACE2-hamster on day 3 post-infection. Figure S3: Human and hamster ACE2 expression in brain sections from an infected hACE2-hamster on day 3 post-infection. Table S1: Scores for clinical signs observed in SARS-CoV-2 infected hACE2-hamsters. Table S2: Experimental studies to develop a COVID-19 model in hACE2-hamsters. References [47,48] are cited in the Supplementary Materials.
