# Supplementary material for: Differences in Susceptibility to SARS-CoV-2 Infection Among Transgenic hACE2-Hamster Founder Lines
Source: Viruses. 2024 Oct 17;16(10):1625. doi: 10.3390/v16101625 (PMC11512293; doi:10.3390/v16101625)
Supplement: Supplementary file 1 [file viruses-16-01625-s001.zip › viruses-3036932-supplementary.pdf]

Supplementary Data

We developed a lethal model for COVID-19 in 8-week-old transgenic hACE2-hamsters. The euthanasia criteria for infected hamsters was based on weight loss (≥ 20% weight-loss cut-off) and/or observation of clinical scores. All animals were observed twice daily for clinical signs as shown in Table S1.

Table S1. Scores for clinical signs observed in SARS-CoV-2 infected hACE2-hamsters

Clinical Signs Observed	Score
Ruffled Fur	**1**
Hunched Position	**1**
Nasal and Ocular Discharge	**1**
Diarrhea	**2**
[1]Lethargy	**2**
[2]Abnormal Gait	**3**
[3]Mild Dyspnea	**3**
[4]Moderate Dyspnea	**5**
[5]Severe Dyspnea	**7**
[6]Severe Diarrhea and/or Abdominal Swelling	**9**

[1]Lethargy: State of unresponsiveness and inactivity.

[2]Abnormal gait: Slow, stiff, or staggered movements, loss of limb flexion or dragging of hind limb.

[3]Mild dyspnea: Increased respiration rate or beginning signs of labored breathing.

[4]Moderate dyspnea: Signs of labored breathing and/or visible gasping for breath.

[5]Severe dyspnea: Drastic decrease in respiration rate and/or increase in gasping for breath (severe difficulty in respiration).

[6]Abdominal swelling: observed in only <2% of animals.

Critical evaluation of the hACE2-hamster founder lines included a series of challenge dose titrations that identified the optimum challenge dose, time of virus shedding, replication in different tissues, and identification of a treatment for use as a positive control in evaluating

potential medical countermeasures. A listing of all experimental studies using hACE2-hamsters to develop a model for COVID-19 is shown in Table S2.

In addition, hACE2 expression levels in tissues from transgenic hamsters were evaluated by RT-PCR and co-localization of human and hamster ACE2 by immunofluorescence (IF) (Figures S1 and S2-S3, respectively), although extensive characterization or mechanistic studies was beyond the scope of our funding.

Materials and Methods

Method for evaluation of hACE2 expression in tissues from transgenic hamsters evaluated by RT-PCR (Figure S1)**.** Tissue samples (lung, liver, brain, heart, kidney, small intestine, and spleen) were harvested from both sexes of hACE2 transgenic and wild-type hamsters. Tissues were placed into the trizol, snap frozen in liquid nitrogen, and stored at -80° until ready for RNA extraction. RNA was prepared by chloroform-ethanol extraction, and quantified using a nanodrop spectrophotometer to verify the quality and quantity of RNA. Primers and probes were obtained from Integrated DNA Technologies (Coralville, IA). For human ACE2, the primer and probe sets were aligned with nucleotides 2571-2597 of human ACE2, which were identical to nucleotides 2265-2291 of pmhyGENIE-3-K18-hACE2, which was used to generate transgenic hamsters. Primers and probes for Human ACE2, 5'GGCTGATTGTTTTTGGAGTTGT-3', 5'-GGATTTTCTCCACTTCTTGCTTT-3' and 5'-/56-FAM/TGGCATTGT/ZEN/CATCCTGATCTTCACTGG/3AIABkFQ/-3' and the hamster ACE2, 5'-TAGGACTGATGTAGGAAGGGTAG-3', 5'-GGAGCAGATGGCTACAACTAT-3' 5'-/56-FAM/TGGCAACCA/ZEN/GTTGATTGAAGATGTGG/3iABkFQ/-3' were used for expression analyses. 2X SensiFAST probe No-Rox one-step kit (Meridian Bioscience, BIO-76005) and Magnetic Induction cycler (Bio Molecular Systems) were used to run the RT-PCR. Regular cycles of 95°C for 3 minutes, and 40 cycles of 60°C for 30 seconds and 95°C for 5 seconds. The results are expressed as the cycle of threshold (cT) and normalized to the ribosomal protein L18 (Rpl18), an internal reference control [1, 2].

Method for co-localization of human and hamster ACE2 in transgenic hamsters evaluated by immunofluorescence (Figures S2-S3). Five µM tissue sections of lung were deparaffinized and rehydrated. Antigen retrieval was achieved by placement of slides in decloaking solution (Dako, Agilent Pathology Solutions, Santa Clara, CA) within a decloaking chamber (Biocare Medical, Pacheco, CA), and processed at 125°C and 20 psi for 4 minutes. The slides were allowed to cool to room temperature, then were exposed to 0.5% Triton for 5 minutes, followed by four washes in phosphate-buffered saline for 5 minutes each. A blocking solution of 10% normal donkey serum, 0.2% Triton, and 0.5% bovine serum albumin in phosphate-buffered saline was applied to the slides for 30 minutes at room temperature. The blocking solution was removed, and the slides were incubated with a rabbit anti-SARS polyclonal antibody (Thermo Fisher), monoclonal human

ACE2 (Invitrogen), or goat-anti-hamster ACE2 (Thermo Fisher) in a blocking solution for 2 hours. Slides were washed three times in phosphate-buffered saline for 5 minutes each, then incubated for 1 hour with donkey secondary antibodies (Invitrogen). For immunofluorescence, a mounting media containing DAPI was applied before the application and sealing of a coverslip. Zeiss LSM 710 microscope was used to capture and process the images using Adobe Photoshop.

Results

Figure S1 shows hACE2 expression in tissues from transgenic hamsters by RT-PCR. Ct values of hACE2 in transgenic hamsters including two from founder 41, and one each from founders 35, 44, and 51 was normalized to the ribosomal protein L18 (Rpl18), an internal reference control [2]. hACE2 mRNA expression was observed in lung, brain, heart, kidney, liver, spleen and small intestine.

Co-localization of human and hamster ACE2 in lungs from an infected hACE2-hamster on day 3 post-infection are shown in Figure S2. IF staining shows that human ACE2 was expressed at low levels in epithelial cells of the lung and SARS-CoV-2 appears to co-localize with both human and hamster ACE2.

Figure S3 shows human and hamster ACE2 expression in brain sections from an infected hACE2-hamster on day 3 post-infection. IF staining shows that patterns of human and hamster ACE2 expression on brain epithelial and ependymal cells (glial cells lining the ventricles) appear to be in different focal areas.

References

Livak K.J., Schmittgen T.D. Analysis of relative gene expression data using real-time quantitative PCR and the 2(− Delta Delta C(T)) Method. Methods, 2001;25:402.

Zivcec M, Safronetz D, Haddock E, Feldmann H, Ebihara H. Validation of assays to monitor immune responses in the Syrian golden hamster (*Mesocricetus auratus*). J Immunol Methods. 2011 May 31;368(1-2):24-35. doi: 10.1016/j.jim.2011.02.004. Epub 2011 Feb 17.

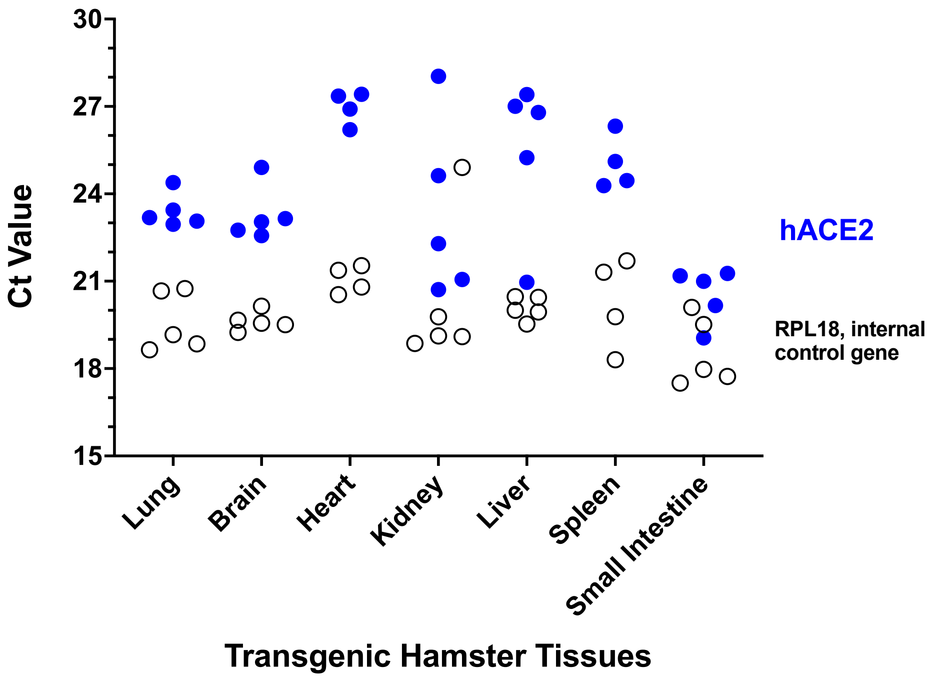

Figure S1. hACE2 expression in tissues from transgenic hamsters by RT-PCR. Ct values of hACE2 in tissues from transgenic hamsters including two from founder 41, and one each from founders 35, 44, and 51. The Ct of hACE2 in transgenic hamsters was normalized to the ribosomal protein L18 (Rpl18), an internal reference control. hACE2 mRNA expression is observed in lung, brain, heart, kidney, liver, spleen and small intestine.

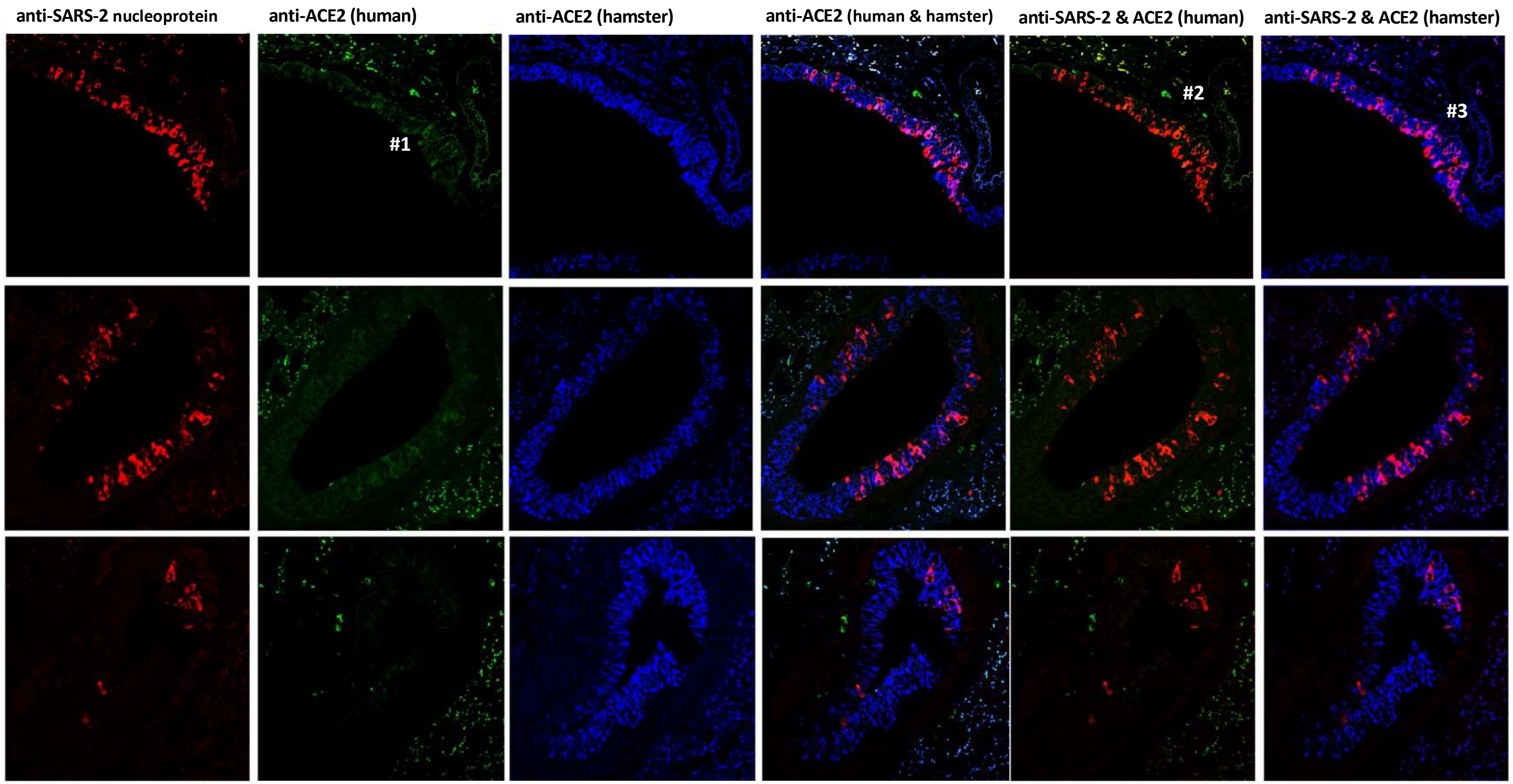

Figure S2. Co-localization of human and hamster ACE2 in lungs from an infected hACE2-hamster on day 3 post-infection. #1 indicates low level immunofluorescent staining of human ACE2. #2 indicates co-localization of SARS-CoV-2 with human ACE2. #3 indicates co-localization of SARS-CoV-2 with hamster ACE2. These data show that human ACE2 is expressed at low levels in epithelial cells of the lung and SARS-CoV-2 appears to co-localize with both human and hamster ACE2.

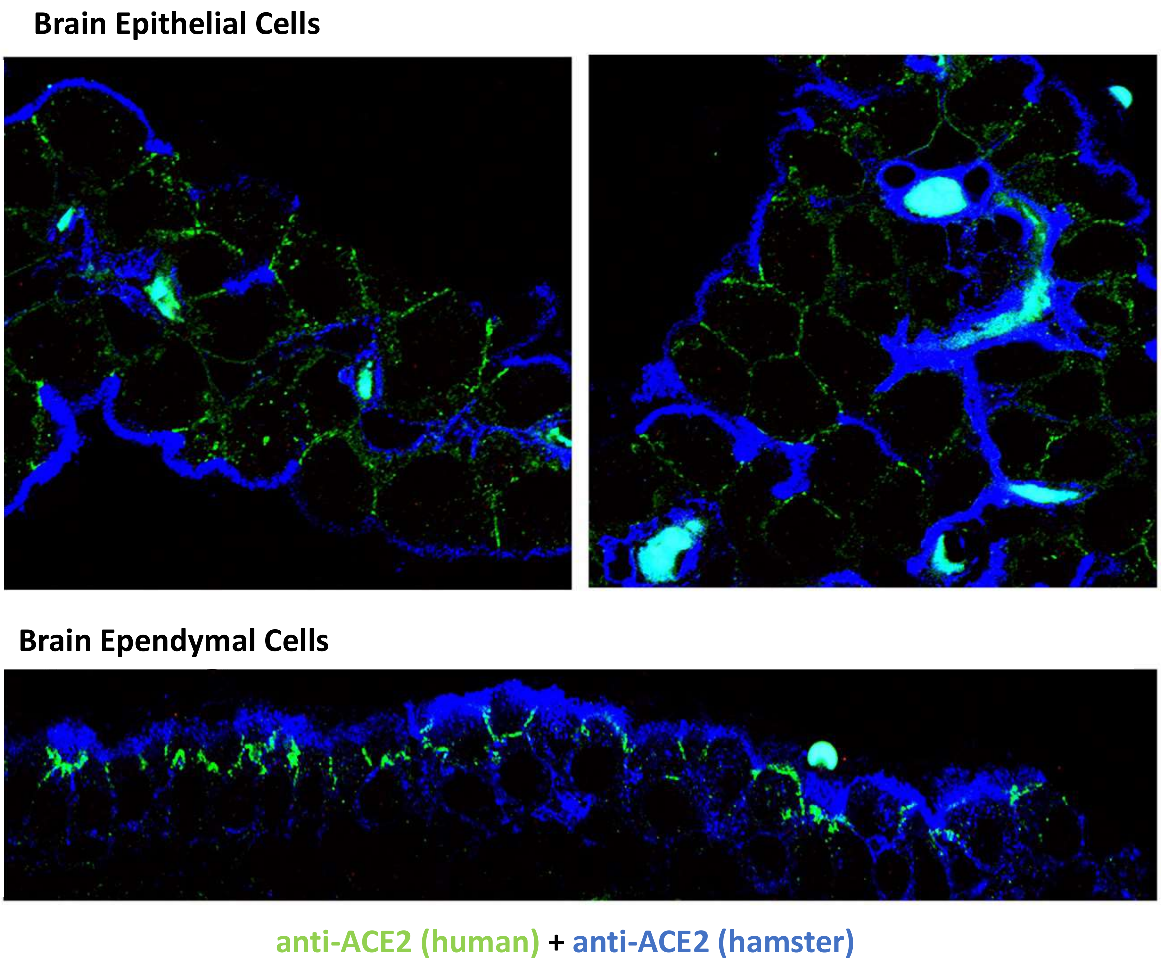

Figure S3. Human and hamster ACE2 expression in brain sections from an infected hACE2-hamster on day 3 post-infection. Immunofluorescent staining show that patterns of human (light green) and hamster (blue) ACE2 expression on brain epithelial and ependymal cells (glial cells lining the ventricles) appear to be in different focal areas.

Table S2. Experimental studies to develop a COVID-19 model in hACE2-hamsters

Study Number	Study Objective	Number of Hamsters	Challenge Dose ($CCID_{50}$ / 0.1 ml)	Experimental Design
NI-1472	SARS-CoV-2 Challenge in F0 hACE2 hamsters	5-6 wk-old<br>7 hACE2 and 7 Wild-Type Littermates	**$10^{4.3}$**	Sac 1 hamster on days 1, 2, 3 post-virus,<br>Collect lungs and trachea for histopath.<br>Oropharyngeal Swabs daily.
NI-1480	Confirmation of SARS-CoV-2 Challenge in F0 hACE2 hamsters	5-6 wk-old<br>6 hACE2	**$10^{4.3}$**	Sac 5 hamsters on day 10 post-virus,<br>Oropharyngeal Swabs for 7 days.<br>Collect lungs and trachea for histopath.
NI-1483	Evaluation of Remdesivir as Treatment for SARS-CoV-2 in F0 hACE2 Hamsters	5-6 wk-old<br>12 hACE2	**$10^{4.3}$**	Body wt & clinical signs for 14 days.
NI-1485	Evaluation of Age-Associated-Susceptibility for SARS-CoV-2 in F0 hACE2 Hamsters	7 “Retired” hACE2 Founders<br>(5 months-old)	**$10^{4.3}$**	Body wt & clinical signs for 14 days.
NI-1488	SARS-CoV-2 Challenge Dose Titration in F1 & F2 hACE2 Hamsters	5-6 wk-old<br>43 hACE2	**$10^{3.3}$, $10^{3.9}$, $10^{4.3}$**	Body wt & clinical signs for 14 days,<br>Oropharyngeal Swabs for 7 days.<br>Sac 3 hamsters on days 2 and 4 for lungs, kidney, spleen, liver, heart, brain, and blood.
NI-1489	Evaluation of GS-441524 as Treatment for SARS-CoV-2 in F1 & F2 hACE2 Hamsters	5-6 wk-old<br>42 hACE2	**$10^{3.3}$**	Body wt & clinical signs for 14 days,<br>Oropharyngeal Swabs for 7 days.<br>Sac 3 hamsters on days 2 and 4 for lungs, kidney, spleen, liver, heart, brain, and blood.
NI-1491	SARS-CoV-2 Challenge Dose Titration in F1 and F2 hACE2-Hamsters	6 wk-old<br>85 hACE2	**$10^{2.3}$, $10^{2.9}$, $10^{3.3}$**	Body wt & clinical signs for 21 days,<br>Oropharyngeal Swabs for 7 days.<br>Sac 3 hamsters on days 2, 3, and 4 for lungs, heart, brain, and blood.

NI-1493	Confirmation of SARS-CoV-2 Challenge in F1 and F2 hACE2-Hamsters	6 wk-old<br>80 hACE2	**$10^{0.3}$, $10^{0.9}$, $10^{1.3}$, $10^{1.9}$, $10^{2.3}$**	Body wt & clinical signs for 21 days,<br>Oropharyngeal Swabs for 7 days.<br>Sac 3 hamsters on days 2 and 4 for lungs, heart, brain, and blood.
NI-1494	Evaluation of Remdesivir, GS-441524, and Poly (I:C) as Treatment for SARS-CoV-2 in hACE2 Hamsters	6 wk-old<br>114 hACE2	**$10^{0.15}$, $10^{0.3}$**	Body wt & clinical signs for 21 days,<br>Oropharyngeal Swabs for 7 days.<br>Sac 3 hamsters on days 2 and 4 for lungs, heart, brain, and blood.
NI-1496	SARS-CoV-2 Challenge Dose Titration in F2 hACE2-Hamsters	7 wk-old<br>94 hACE2	**$10^{0.3}$, $10^{0.9}$, $10^{1.3}$, $10^{1.9}$, $10^{2.3}$, $10^{2.9}$, $10^{3.3}$, $10^{3.9}$, $10^{4.3}$**	Body wt & clinical signs for 21 days.<br>Oropharyngeal Swabs for 7 days.
NI-1498	SARS-CoV-2 Challenge Dose Titration in Four hACE2 Hamster Founder Lines	8 wk-old<br>204 hACE2	**$10^{0.15}$, $10^{0.3}$, $10^{0.9}$, $10^{4.3}$**	Body wt & clinical signs for 21 days.<br>Oropharyngeal Swabs for 7 days.<br>Sac 4 hamsters on day 21 post-virus for lungs, heart, brain, and blood.
NI-1499	Evaluation of SARS-CoV-2 Replication Kinetics and Pathology in Four hACE2 Hamster Founder Lines	8 wk-old<br>100 hACE2	**$10^{0.3}$**	Body wt & clinical signs for 21 days,<br>Oropharyngeal Swabs for 5 days.<br>Sac 3 hamsters on days 2, 4, 6, and 21 for lung, brain, heart, kidney [with adrenals], liver, spleen, eyes, stomach, duodenum [with pancreas], jejunum, ileum, cecum, colon, thigh muscle, femur, ovaries, testes, trachea, and nasal turbinates.
NI-1500	Evaluation of Poly (I:C) Treatment for SARS-CoV-2 in hACE2-hamsters	8 wk-old<br>20 hACE2	**$10^{0.3}$**	Body wt & clinical signs for 21 days.<br>Oropharyngeal Swabs for days.
NI-1502	Evaluation of Dexamethasone and Poly (I:C) Treatment for SARS-CoV-2 in hACE2-Hamsters	8 wk-old<br>121 hACE2	**$10^{0.3}$**	Body wt & clinical signs for 21 days,<br>Oropharyngeal Swabs for 5 days.<br>Sac 5 hamsters on days 10 and 12 for lungs, heart, brain, and blood.
